# Food and Nutrient Intakes of Jamaican Immigrants in Florida

**DOI:** 10.1007/s10903-018-0770-1

**Published:** 2018-06-27

**Authors:** C. R. Oladele, Sangita Sharma, Jimin Yang, Elizabeth B. Pathak, David Himmelgreen, Getachew Dagne, Wendy Nembhard, Thomas Mason

**Affiliations:** 1 Equity Research and Innovation Center, Yale School of Medicine, Yale University, 100 Church St. South (Suite A200), PO Box 208093, New Haven, CT 06519 USA; 2 Center for Neuroepidemiology and Clinical Neurological Research, Yale School of Medicine, Yale University, New Haven, CT USA; 3 Department of Medicine, University of Alberta, Edmonton, AB Canada; 4 Health Informatics Institute, University of South Florida, Tampa, FL USA; 5 Morsani College of Medicine, University of South Florida, Tampa, FL USA; 6 Department of Anthropology, University of South Florida, Tampa, FL USA; 7 Department of Pediatrics and Arkansas Children’s Hospital Research Institute, University of Arkansas for Medical Sciences, Little Rock, AR USA; 8 Department of Epidemiology and Biostatistics, University of South Florida, Tampa, FL USA; 9 Department of Environmental and Occupational Health, University of South Florida, Tampa, FL USA

**Keywords:** Nutrient intakes, Immigrants, Caribbean, Diet

## Abstract

This study assessed dietary intakes, nutritional composition, and identified commonly eaten foods among Jamaicans in Florida. Dietary intake was assessed among 44 study participants to determine commonly eaten foods and nutrient composition. Weighed recipes were collected and analyzed to determine nutrient composition for traditional foods. Top foods that contributed to macronutrient and micronutrient intake were identified and adherence to dietary recommendations was evaluated. Mean daily energy intake was 2879 (SD 1179) kcal and 2242 (SD 1236) kcal for men and women respectively. Mean macronutrient intakes were above dietary recommendations for men and women. Top foods contributing to energy included rice and peas, sweetened juices, chicken, red peas soup, and hot chocolate drink. Results showed sodium intake was more than double the adequate intake estimate (1300–1500 mg). Findings highlight the need to include commonly eaten traditional foods in dietary questionnaires to accurately assess diet-related chronic disease risk. Findings have implications for risk factor intervention and prevention efforts among Jamaicans.

## Background

Information on the dietary intakes of English-speaking Caribbean immigrants in the US is scarce, despite the growing number of persons emigrating to the US from the Caribbean region [1, 2]. Current methods of assessing the dietary intakes of the US population do not permit adequate stratification of ethnic sub-populations including Caribbean immigrants. Further, current methods lack the ability to accurately assess the dietary composition of ethnically diverse subpopulations due to the paucity of information on the nutrient intakes of ethnic foods [3, 4]. This is particularly true for African-descent Caribbean immigrants for whom there is no existing information on their dietary intakes. Knowledge on the dietary intakes of this and other subpopulations is essential as it helps to determine risk for chronic diseases and can inform public health prevention efforts.

The South Florida region is one of many regions along the eastern seaboard that is a top destination for Caribbean immigrants, particularly Jamaican immigrants. There are an estimated 106,000 Jamaicans who live in the South Florida region, [5] yet little is known about their dietary intakes which contribute to development of chronic diseases. Evidence from a range of migrant groups show that immigrants initially have a favorable dietary profile which deteriorates with increased length of time spent in the US [6–8]. It was therefore imperative to understand the intakes of this population for whom information is scarce, and among whom diet-related diseases such as hypertension and diabetes are prevalent [9–11]. This study presents a comprehensive description of the nutrient and food intakes of Jamaican immigrants in the US, describes the level of adherence to dietary recommendations, [12] and discusses implications for the prevention of chronic disease.

## Methods

### Participants

Participants were recruited for participation in the Jamaicans Migrating to the US Study (JAMUS) from local churches and community organizations in South Florida. A 2-stage cluster sampling approach was used to identify potential participants. Churches and organizations were randomly selected from an enumeration list and approached for participation. Eight organizations (47%) agreed to participate and provide member rosters to facilitate random selection of individuals for recruitment. Persons were eligible for participation if they (1) were born in Jamaica, (2) self-identified as black, (3) were 25–64 years, (4) had residence in the US for a year, and (5) did not leave the US for more than 30 consecutive days in the past year. Persons randomly selected from member rosters were contacted by telephone to introduce the study and obtain consent for participation. Those who agreed to participate were scheduled for an in-person interview at local libraries or a place of their choosing. The interviewer obtained signed informed consent from participants prior to the start of the interview. The overall response rate for the study was 64 percent. The final sample included 91 participants across eight organizations and churches, meeting the minimum sample size required (n = 80) to address the larger study aims. The current analysis includes 45 of 91 participants who completed 24-h recalls in the JAMUS study.

### Data Collection

Participants underwent in-person interviews conducted by the investigator (CO). Participants were administered a 24-h dietary recall and health survey, that included demographic, anthropometric (e.g. weight, height), and lifestyle questions (e.g. physical activity, smoking). This study was approved the University of South Florida Institutional Review Board (protocol # 00002078).

### 24-h Recall

Information on dietary intakes was obtained from 24-h recalls to (1) estimate nutrient intakes in a sample of Jamaican immigrants and (2) determine foods for inclusion on a quantitative food frequency questionnaire. Single interviewer-administered 24-h recalls were used to estimate food and nutrient intakes using the multi-pass 24-h recall method. All interviews were conducted by the first author who had extensive training in administering 24-h recalls. A procedural script was developed to increase standardization of interviews. During interviews, participants were asked to recall all food and drink consumed on the previous day. Participants were asked to provide complete food descriptions including information on preparation methods, additions/toppings, and portion size. Culturally appropriate food models and common kitchen utensils were used to help participants accurately estimate portion sizes of their reported intakes. Participants were then probed for easily forgotten food and drink such as snack foods, alcohol, candy, and supplements. The interview concluded with a summary of foods listed, portion sizes, and details of the foods reported eaten. Recalls were administered on weekdays and weekends to account for differences in intakes across days of the week [13, 14].

### Data Analyses

Data from recalls were entered in the Nutrition Data System for Research (NDSR) for foods that were included in the software [15]. The NDSR is a dietary analysis program designed for the collection and analysis of 24-h dietary recalls and the analysis of records, menus, and recipes. Data on the nutritional composition of traditional Jamaican dishes were obtained from prior work by Sharma et al. [16, 17]. The nutrient content of other reported foods was obtained from the food composition information in the NDSR. Self-reported portion size was used to accurately estimate total energy. Each food item was quantified as reported by participants and weighed to obtain the gram weight for inclusion in the nutrient analysis. The weight of each food and drink portion was entered in the NDSR along with information on ingredients for the nutrient analysis. Nutrient values were calculated for all foods entered in the software and then exported to SAS [18] to conduct additional analyses. Nutrient values did not include the use of dietary supplements.

We calculated the percentage of the sample population that fell below and above Dietary Recommended Intakes to determine excess and inadequate nutrient intakes. Binomial tests were conducted to test differences in the percent of the sample population that fell below and above the DRIs by sex. To examine the top 10 foods that contributed to energy and macronutrient intake we combined alcoholic beverages, crackers, hot chocolate drinks, juice, green salads, and bread into categories. Other food and drink were kept as individual items in analyses including mixed dishes (e.g. one pot soups, brown stew chicken). Top foods that contributed to macronutrient intake were determined by summing nutrient totals for each food reported and ranking foods. The same process was used to determine top foods contributing to energy.

## Results

Data from 44 participants were included in the final analysis. Data for one participant who was on a weight gain diet and had extreme nutrient values was excluded. Table 1 presents the demographic characteristics of the sample. The sample included a similar number of men and women. Participants lived in the United States for an average of 19 years and the mean age of migration for participants was 27 years. Mean body mass index was high at 29.7 kg/m^2^ which suggests that on average, participants were overweight [19].

**Table 1:** Jamaicans migrating to the United States (JAMUS) participant demographics

Characteristic	Total	Men	Women
	n = 44	n = 23	n = 21
Mean age (years, SD)	48 (10.2)	49 (9.7)	48 (11.0)
Mean age at migration (years, SD)	27 (12.5)	28 (10.2)	26 (15.0)
Mean years of stay in US (years, SD)	19.3 (9.4)	18.4 (9.1)	20.0 (9.8)
Reside in ethnic enclave	24.4	12.5	38.1
Marital status			
Married (%)	65.9	78.3	52.4
Single (%)	25.0	17.4	33.3
Divorced (%)	9.1	4.3	14.3
Education level			
College and beyond (%)	81.8	78.3	85.7
High school graduate (%)	15.9	17.4	14.3
0–11 years education (%)	2.3	4.3	0
Employed (%)	90.9	52.2	61.9
Mean Body Mass Index (kg/m^2^) SD	29.7 (6.3)	29.6 (5.9)	30.0 (6.8)

*SD* standard deviation

Table 2 presents a comparison of mean daily nutrient intakes for men and women with dietary recommendations. Overall, results showed mean intakes well above dietary recommendations for men and women, except fiber and vitamin E. Mean daily intake of carbohydrates and fats was more than double and protein intake was double that of the recommended intakes for these nutrients. Mean daily saturated fat intake was 33 and 29 g for men and women respectively. Intake of vitamins A, C, B-12, and folate were also above the recommendation for intakes. Potassium intake among men was consistent with recommendations while intake among women was above the recommended adequate intake level (3193 vs. 2800). Sodium intake was above the recommended level of intake for men and women. Results showed mean sodium intake was 4341 and 3362 mg respectively for men and women compared to the recommendation of 1.5 mg. Iron intake was higher than the dietary reference intake among men (19 vs. 8 mg) and among women 51–70 years (17 vs. 8 mg).

**Table 2:** Adherence to dietary reference intakes among JAMUS study participants

	Men			Women		
	Mean	SD	DRI	Mean	SD	DRI
Energy (kcal)	2879	1179	NDRA	2242	1236	NDRA
Protein (g)	112	54	19–70: 56	89	44	19–70: 46 g
Carbohydrates (g)	374	237	19–70: 130	310	167	19–70: 130
Sugars (g)	166	166	NDRA	118	99	NDRA
Total dietary fiber (g)	24	18	19–50: 38 51–90: 30	25	15	19–50: 25 51–70: 21
Fat (g)	100	38	19–70: 20–35	76	56	19–70: 20–35
Saturated fat (g)	33	15	NDRA	29	25	NDRA
Vitamin B-6 (mg)	2.6	1.5	19–50: 1.3 51–70: 1.7	2.3	1.0	19–50: 1.3 51–70: 1.5
Folate, DFE (mcg)	529	347	19–70: 400	467	263	19–70: 400
Vitamin B-12 (mcg)	5	4.0	19–70: 2.4	5.0	5.0	19–70: 2.4
Vitamin C (mg)	159	141	19–70: 90	160	144	19–70: 75
Vitamin E (mg)	9	6.0	19–70: 15	12	20	19–70: 15
Calcium (mg)	806	585	19–70: 1000	618	369	19–70: 1000
Iron (mg)	19	9.0	19–70: 8	17	9.0	19–50: 18 51–70: 8.0
Zinc (mg)	13	7.0	19–70: 11	11	7.0	19–70: 8.0
Selenium (mcg)	179	106	19–70: 55	127	74	19–70: 55
Magnesium (mg)	390	191	19–30: 400 31–70: 420	364	232	19–30: 310 31–70: 320
Sodium (mg)	4341	2330	19–50: 1500 50–70: 1300	3362	1885	19–50: 1500 50–70: 1300
Potassium (mg)	3778	1834	19+: 3800	3193	1682	19+: 2800
Omega-3 fatty acids (g)	2.5	1.6	19–70: 0.6–1.2	1.8	1.5	19–70: 0.6–1.2

*DRI* dietary reference intakes

*SD* standard deviation

To better understand intakes, we examined the percentage macronutrient contribution to total energy (data not shown) and the percentage contribution of foods to energy. Results showed carbohydrate intake accounted for the highest percentage contribution to total energy (52%) followed by saturated fat (35%), total fat (31%), protein (16%), and alcohol (1%). Top 10 foods that contributed to total energy intake are presented in Table 3. Rice and peas, sweetened juices, chicken, and red peas soup, were among the top foods contributing to energy among study participants. Rice and peas made the highest contribution to energy (8.0%) followed by sweetened juices (5.9%) and chicken (4.5%). Red peas soup, a traditional one pot meal that includes meat, vegetables, and dumplings, contributed 2.8 percent to energy. Other foods included milo, white rice, fish, pork, and ackee and saltfish. The latter is the national dish of Jamaica that is commonly eaten on weekends and holidays.

**Table 3:** Top ten foods that contributed to energy intake

Food reported dietary during recall	% Contribution to daily energy intake
Rice and peas^a^	8.0
Sweetened juices	5.9
Baked chicken	4.5
Red peas soup^b^	2.8
Hot chocolate/milo	2.7
Crackers	2.6
White rice	2.4
Whole wheat bread	2.0
Fish	1.6
Pork	1.4
Total	33.9

^a^A mixed dish of white rice and gungu (pigeon) peas (or kidney beans). Coconut milk, onions, scallions, salt, pepper, and thyme are added

^b^A thick stock soup made with red peas (kidney beans), pig tail or salted beef, yam, sweet potato, spinners (dumplings), thyme, and scallion

The top 10 foods that contributed to nutrient intakes are presented in Table 4. Top foods contributing to protein included chicken, rice and peas, red peas soup and ackee and saltfish, all of which contributed most to energy intake. Sweetened juice was the top contributor to carbohydrate intake accounting for almost 11% followed by rice and peas which accounted for 10.4%. Foods that contributed both to fat and saturated fat included rice and peas, chicken, butter, macaroni and cheese, ox tail, and red peas soup. Dessert items such as cheesecake, cookies, and breadsticks were also top contributors to fat intake. Results for fiber showed that grain/lentil-based dishes or foods contributed most to fiber intake.

**Table 4:** Top 10 foods contributing to nutrient intake

#	Protein		Carbohydrate		Fat		Saturated fat	
	Food	% Contribution to protein	Food	% Contribution to carbohydrate	Food	% Contribution to fat	Food	% Contribution to saturated fat
1	Baked chicken	13.6	Sweetened juices	10.9	Baked chicken	6.8	Rice and peas^a^	12.9
2	Fish	8.5	Rice and peas^a^	10.3	Rice and peas^a^	5.4	Baked chicken	5.2
3	Rice and peas^a^	4.9	Hot chocolate/milo	4.8	Crackers	3.7	Butter	4.6
4	Red peas soup^b^	4.0	White rice	3.3	Pork	3.2	Red peas soup^b^	3.9
5	Pork	2.7	Syrup	2.6	Red peas soup^b^	3.2	Macaroni and cheese	3.4
6	Curried goat^d^	2.7	Boiled plantain	2.6	Peanuts	2.9	Pork	3.4
7	Whole wheat bread	2.4	Whole wheat bread	2.6	Butter	2.5	Crackers	2.2
8	Ackee and saltfish^e^	2.0	Crackers	2.4	Ackee and saltfish^e^	2.4	Oxtail^f^	2.2
9	Macaroni and cheese	1.7	Red peas soup^b^	2.2	Macaroni and cheese	2.1	Cheesecake	1.9
10	Sandwich or wrap	1.5	Banana	2.0	Oxtail^f^	1.9	Cookies	1.9
Total		43.9		43.7		34.2		41.6

#	Fiber		Sodium		Potassium		Sugar	
	Food	% Contribution to fiber	Food	%	Food	%	Food	%
1	Rice and peas^a^	8.1	Rice and peas^a^	10.9	Sweetened juices	5.4	Flavored drink/juice	24.0
2	Red peas soup^b^	5.8	Baked chicken	5.2	Rice and peas^a^	5.2	Brown sugar	10.0
3	Whole wheat bread	4.4	Red peas soup^b^	4.8	Hot chocolate/milo	4.6	Jamaican drink syrup	8.0
4	Pear	3.4	White rice	4.3	Red peas soup^b^	3.9	White sugar	3.7
5	Stew peas^c^	3.4	Fish	4.0	Boiled plantains	3.8	Banana	3.1
6	Banana	3.2	Crackers	2.9	Fish	3.5	Cake	3.0
7	Boiled plantain	2.7	Whole wheat bread	2.5	Coconut water	3.1	Soda	2.6
8	Garbanzo beans	2.4	Stew peas^c^	2.2	Baked chicken	3.1	Homemade fruit punch	2.3
9	Coconut water	2.0	Pizza	2.1	Banana	3.1	Fruit salad	2.1
10	Sweetened juices	1.9	Oxtail^f^	1.9	Clam chowder	2.0	Hot chocolate	1.0
Total		37.4		40.8		37.7		59.8

^a^A mixed dish of white rice and gungu (pigeon) peas (or kidney beans). Coconut milk, onions, scallions, salt, pepper, and thyme are added

^b^A thick stock soup made with red peas (kidney beans), pig tail or salted beef, yam, sweet potato, spinners (dumplings), thyme, and scallion

^c^Red peas or kidney bean stew cooked with salted meats (beef or pigs tail) garlic, scallion, thyme, coconut milk (optional) and spinners

^d^Fried goat cooked down with onions, curry powder, pimento, scallion, thyme, and other seasoning

^e^A fried dish cooked with saltfish, vegetables and Ackee, an oily fruit (National dish of Jamaica)

^f^Oxtail is boiled with butter beans, onions, scallion, thyme, boiled down to a thick sauce (Food Descriptions adapted from Sharma et al. [37])

We also examined micronutrients that are associated with prevalent risk factors (i.e. hypertension and diabetes) in the target population. Results were consistent with foods that contributed most to macronutrient intake. Rice and peas was the top source of sodium intake while sweetened juices and pear (avocado) were top foods for potassium intake. Flavored juice/drink was the top source of total sugar intake (24%) among participants (Table 4).

## Discussion

This study assessed the dietary intakes of Jamaican immigrants in the US to describe food and nutrient intakes and make comparisons to dietary recommendations. Results showed that Jamaican immigrants consume traditional foods frequently despite living the US for an average of 19 years. Traditional foods were also shown to be top contributors to energy and macronutrient intakes. The top foods identified are consistent with foods that were commonly seen on menus at Jamaican restaurants in the two counties. Findings showed that study participants had intakes well above dietary recommendations.

Macronutrient intakes were higher than recommended which suggests that total energy intakes are above the recommendation based on individual characteristics. Carbohydrate intake is of concern given higher than recommended intakes in a population with high diabetes prevalence. The higher than recommended macronutrient intakes may result from large portion sizes that are embedded in cultural norms. Greater affordability of foods post migration, particularly meats, may also explain the observed higher than recommended macronutrient and energy intakes. However, this study did not assess pre-migration dietary habits.

Our findings also highlighted excess intake of sodium and food sources of sugar intake that are high in added sugar. The top foods that contributed to sodium were like the top foods (i.e. traditional mixed food dishes) that contributed to energy and macronutrient intake, most of which were unprocessed, non-fast foods. Though traditional mixed food dishes are not typically considered fast food, they are readily available at small ethnic restaurants. Dishes prepared at restaurants are often higher in fat and sodium content than when prepared in home [20, 21]. Participants may also prepare traditional dishes at home that are high in sodium content, similar to restaurants, which contribute to excess sodium intake. This contrasts findings among other immigrant groups that show fast and processed foods as main sources of sodium intake [22, 23]. Food and drink sources of sugar intake are consistent with findings from national studies that show beverages as main sources of added sugars [24–26]. Similar results have also been shown among Caribbean populations in region [27]. Iron intakes were also above the dietary recommendation. This may be due to increased legume consumption, which is consistent with frequently eaten foods (e.g. red peas soup, rice and peas). Our study findings highlight the need for nutrition intervention programs to improve overall intakes in this target population. There is also a need for specific focus on sodium and sugar intake given the established association with diabetes and hypertension, both prevalent risk factors among Jamaicans [28, 29].

Future research will investigate cultural perceptions of fast food among Jamaicans and perceptions of risk for diet-related diseases. We will also reexamine intakes in a larger sample and examine associations with chronic disease risk.

### Study Limitations

Our study provides foundational work to support future nutrition research among Jamaican and other Caribbean immigrants. Though foundational, our study had several limitations. Our sample primarily comprise Jamaicans who have higher educational attainment despite recruitment efforts to ensure adequate socioeconomic representation. Findings may not be generalizable to lower educated individuals whose dietary intake may differ. Our results may underestimate nutrient intakes in this population based on prior research that show healthier dietary intakes among more educated persons [30–32]. Determination of usual food and nutrient intakes was based on single administered dietary recalls. Though single administration precluded the ability to estimate the distribution of usual intakes, it was sufficient to achieve our study goal of estimating mean usual intakes and identifying frequently consumed food and drink at the population level [33]. To our knowledge, this is the first food and nutrient assessment conducted specifically among Jamaican immigrants in the US. Finally, total energy estimates may be underestimated since they are based on single dietary recalls and may not fully capture intra-individual variation [34]. It is also possible that participants under- and over-reported intakes which may have impacted total energy estimates. Since this was not a primary goal of our study, we did not conduct further testing to validate energy estimates.

### New Contributions to the Literature

Our study describes the food and nutrient intakes of Jamaican immigrants in Florida, US. We examined foods that contributed most to nutrient intakes and showed that traditional food sources account for most intakes. Study participant diets mainly consisted of poultry, fish, legumes, rice, and fats from a variety of sources. Foods such as rice and peas, oxtail, pork, one pot soups, and other traditional meat dishes unique to Jamaican culture, were significant sources of energy and nutrient intakes. Top food sources that contributed to total energy were consistent with the carbohydrate-rich diet observed in many Caribbean countries including Jamaica.

Similar studies in this population were conducted in England and are now more than 10 years old; this study provides contemporaneous data on Jamaican immigrants in a US context, which has not been previously done [16]. Comparison of findings from prior studies varied in consistency with the current study findings. The distribution of the percent contribution of macronutrients to energy differed from prior findings among Caribbean persons (mostly Jamaicans) in England [16]. Caribbean persons in England had lower mean macronutrient intakes compared to persons in the current study sample [16, 35]. The percent contribution to energy from fat, saturated fat, and alcohol was higher compared to current study results. In contrast, a 2014 study showed lower percent contribution to energy from saturated fat and carbohydrates [35]. When we compared foods, results were consistent with prior findings which showed that rice and peas, chicken, macaroni and cheese, and one-pot soups were top contributors to energy [35–38]. Findings among Jamaicans in England also showed rice and peas and chicken as main sources of energy intake. Other foods, however, differed from those found in the current study. Foods such as hardough bread, dumplings, condensed milk and yam were main sources of energy among Jamaicans in England [37]. Comparisons to US blacks showed Jamaican immigrants in our study have higher mean energy and sodium intakes [39].

In examining nutrient and food intakes among Jamaican immigrants, we identified several nutrients for intervention for the prevention of cardiovascular disease. In examining protein, carbohydrate, and total fat intake, results indicate the need for a reduction in total calories. Additionally, our findings showed that intake of micronutrients that confer cardiovascular benefit are low, while intake of micronutrients associated with increased cardiovascular risk (e.g. sodium-rich foods and flavored drinks/juice) was higher than recommended.
